# Assessment of Fractal Synchronization during an Epileptic Seizure

**DOI:** 10.3390/e26080666

**Published:** 2024-08-05

**Authors:** Oleg Gorshkov, Hernando Ombao

**Affiliations:** Statistics Program, King Abdullah University of Science and Technology, Thuwal 23955, Saudi Arabia; hernando.ombao@kaust.edu.sa

**Keywords:** the Hurst exponent, synchronization, epileptic seizure, fractals, generalized variance

## Abstract

In this paper, we define fractal synchronization (*FS*) based on the idea of stochastic synchronization and propose a mathematical apparatus for estimating *FS*. One major advantage of our proposed approach is that fractal synchronization makes it possible to estimate the aggregate strength of the connection on multiple time scales between two projections of the attractor, which are time series with a fractal structure. We believe that one of the promising uses of *FS* is the assessment of the interdependence of encephalograms. To demonstrate this approach in evaluating the cross-dependence between channels in a network of electroencephalograms, we evaluated the *FS* of encephalograms during an epileptic seizure. Fractal synchronization demonstrates the presence of desynchronization during an epileptic seizure.

## 1. Introduction

One of the important issues of neurophysiology is the identification and characterization of various dynamic states during epileptic seizures, which will be critical for understanding the mechanism of initiation, spread, and termination of ictal activities. One of the recently developed tools for solving this problem is the study of synchronization between neuronal populations [1,2,3]. Synchronization between neuronal populations is an important mechanism for establishing functional connections between various regions of the brain [4]. The process of neuronal synchronization occurs through the transmission of excitatory and inhibitory impulses [4,5,6]. One of the manifestations of the phenomenon of synchronization between neuronal populations in the brain is the process of recognition of an incoming pattern, which occurs due to the mutual influence of neurons [6]. Timofeev et al. [2] differentiated between two types of neuronal synchronization: long-range and local synchronization. Long-range synchronization is detected using electrodes located at some distance apart. The local (short-range) synchronization can be discovered using electrodes placed at short (less than 1 mm) distances from each other. Timofeev et al. [2] revealed that during an epileptic seizure, there is a decrease in the long-range synchronization and an increase in the local synchronization. According to [2], this is a consequence of a reduction in neural firing ability and a decrease in transmitter release. However, it should be taken into account that neural signals are constantly combining and recombining in order to create suitable synchronization patterns on different time scales. It is believed that such processes allow the brain to be more flexible in making decisions on different time scales [7,8]. Hutchison et al. [7] concluded that the brain must dynamically unite, synchronize, and react to internal and external stimuli across various time scales. Our goal in this paper is to develop a new method for estimating the aggregate strength of connectivity (aggregate interdependence) between populations of neurons at multiple time scales. One of the tools to solve this problem is fractal analysis, which enables one to analyze the signal structure at different time scales, extracting from it a common structural substrate characterizing the self-similarity of the signal at different time scales. Applying the basics of fractal analysis will allow us to develop a new method for assessing the aggregate interdependence between self-similar characteristics of signals on multiple time scales, which we will define below as fractal synchronization.

Schiff et al. [1] demonstrated that there are differences between the initiation and termination dynamics of ictal activities, which allowed them to distinguish the initial and termination periods of an epileptic seizure. Further research revealed different types of neuronal synchronization inherent in the initial and termination periods of epilepsy. In [9,10], the initial period of a seizure was demonstrated to be characterized by neuronal desynchronization. Conversely, the termination period is characterized by neuronal synchronization (or a return from a desynchronized to a synchronized state). Indeed, ref. [3] showed that desynchronization decreases from the initiation period, which then reaches its minimum at the termination of the seizure period [1,11]. Schindler et al. [11] reported that desynchronization is characterized by an increase in amplitude (power) of high-frequency components (gamma band: 30–50 Hz) [12] of EEG signals. This result is consistent with conclusions in [13,14], which show that there is an increase in high-frequency components in the initial period of a seizure. At the same time, Schindler et al. [11] pointed out that an increase in power in low-frequency components (delta band, theta band, alpha band: 0.5–12 Hz) [12] is inherent in synchronization to enable a contemporaneously occurring refractory state in the termination period. Thus, for correct estimation of neuronal synchronization during an epileptic seizure, the method used should be sensitive to the amplitude–frequency characteristics of the EEG signal, which will allow for distinguishing the initial and termination period of an epileptic seizure. Our proposed method is based on fractal analysis;one of the tools used is the Hurst exponent. It is shown below that the Hurst exponent is a good indicator of the power of the high-frequency or low-frequency component of the signal. Thus, the proposed method of fractal synchronization allows taking into account not only the fractal structure of the EEG signal but also its amplitude–frequency characteristic, which is an important factor characterizing the initial and termination period of an epileptic seizure.

In [5], the concept of synchronization is proposed as “more or less synonymous with interdependence”. There are various methods for assessing synchronization (or quantifying the degree of synchronization between coupled systems). The most known approaches to assessing the degree of synchronization are linear cross-correlation [15], mutual information [16,17], phase synchronization [15,18], and generalized synchronization [19,20]. In [12], a general framework is shown for assessing the synchronization between oscillatory components in a multivariate system. Here in this paper, our proposed method is based on the idea of fractal synchronization, evaluating the interdependence between two signals, which are projections of some attractor, on multiple time scales.

The idea of generalized synchronization has become very popular for the analysis of the interdependence of EEG signals [21] since it allows one to reveal a non-linear relationship (coupling) between signals using elements of chaos theory [22]. One important property of generalized synchronization is that the signals analyzed do not have to be similar to each other [20]. The idea of generalized synchronization was proposed by Rulkov et al. [19]. Generalized synchronization is characterized by the existence of a continuous functional relation:

(1) $$\vec{r}(t)=\vec{\vartheta }(\vec{d}(t)),$$

where $\vec{\vartheta }$ is a continuous function; $\vec{r}(t)$ is the chaotic trajectory reconstructed in the embedding space ${\vec{R}}_{E}$ from scalar variables taken from the response system; $\vec{d}(t)$ is the chaotic trajectory reconstructed in the embedding space ${\vec{D}}_{E}$ from scalar variables taken from the drive system. There is no unique form of $\vec{\vartheta }$ that characterizes dependence. However, one potential form can be derived from the mutual information between a pair of signals (or between many signals) from the system (see [19]), which may reveal a non-linear relationship between the signals. One advantage of generalized synchronization is that this approach can be applied in practice to analyze chaotic synchronization in real physical systems. However, this idea is a particular case of the more general idea of stochastic synchronization formulated by Afraimovich et al. [23] for various forms of coupling. Consider the idea of stochastic synchronization, which allows us to introduce the definition of fractal synchronization, which is a special case of stochastic synchronization.

The mathematical definition of stochastic synchronization is proposed in [23]. Let

(2) $$\frac{d{\vec{x}}_{1}}{dt}={\vec{F}}_{1}({\vec{x}}_{1}),\;\frac{d{\vec{x}}_{2}}{dt}={\vec{F}}_{2}({\vec{x}}_{2}),\;{\vec{x}}_{1}\in R^{m},\;{\vec{x}}_{2}\in R^{n}$$

be systems defining the first and second self-excited oscillators. They explored the system

(3) $$\frac{d{\vec{x}}_{1}(t)}{dt}={\vec{F}}_{1}({\vec{x}}_{1})+c{\vec{f}}_{1}({\vec{x}}_{1},{\vec{x}}_{2}),\frac{d{\vec{x}}_{2}(t)}{dt}={\vec{F}}_{2}({\vec{x}}_{2})+c{\vec{f}}_{2}({\vec{x}}_{1},{\vec{x}}_{2})$$

If system (3) loses its dependence on initial conditions when time tends to infinity, namely, the set of initial states corresponds to the same final points, and then the set of such points is called an attractor of the system. Thus, an attractor implies a preferred trajectory of a temporally evolving system. Stochastic synchronization occurs for values of the coupling parameter in the interval $c_{1}<c<c_{2}$ if, for these values of *c*, system (3) generates an attractor $A_{c}$ such that the projection of the attractor $A_{c}$ on the individual subspace $A_{1}$ can be translated to another projection of the attractor $A_{c}$ on the individual subspace $A_{2}$ with the help of a mutually unambiguous, mutually continuous function *g*:

(4) $$A_{2}=g(A_{1})$$

That is, there is a homeomorphous function *g* by means of which points on the individual subspace $A_{1}$ are mutually unambiguously matched to the points on the individual subspace $A_{2}$. Thus, the mapping preserves ranks of distances: if $\left|x_{1}-x_{2}\right|<\left|x_{3}-x_{4}\right|$, then $\left|g(x_{1})-g(x_{2})\right|<\left|g(x_{3})-g(x_{4})\right|$.

Further research on stochastic synchronization led to the conclusion that noise can influence the dynamics of the system, causing or enhancing synchronization [24,25]. C. Pang et al. [26] suggest that stochastic synchronization plays an important role in network integration, which is the basis for brain functioning. In particular, they revealed that stochastic synchronization is related to the hierarchy of neural timescales inherent to the brain. However, this general framework of stochastic synchronization is difficult to implement into a practical algorithm for analyzing synchronization between neuronal populations (or other biological systems). Therefore, to implement a practical algorithm, we limit ourselves to considering a special case of stochastic synchronization, namely fractal synchronization. Next, let us consider the idea of topological synchronization [27,28], the properties of which allow for introducing the concept of fractal synchronization. In [27], the concept of topological synchronization is introduced for which condition (4) holds. The topological synchronization theorem in [27] states that projections of the attractor on the individual subspaces have the same Hausdorff dimensions ($D_{H}$) at least:

(5) $$D_{H}(A_{1})=D_{H}(A_{2})$$

The Hausdorff dimension $D_{H}$ can be defined as:

(6) $$D_{H}\propto -\frac{\log N_{H}}{\log\Omega }$$

where $N_{H}$ is the number of objects of characteristic size Ω needed to cover the attractor. If the Hausdorff dimension $D_{H}$ of the attractor is not an integer, then such an attractor has a fractal structure. The Hausdorff dimension $D_{H}$, or more specifically, fractal dimension, is connected with the Hurst exponent H by relation [29]:

(7) $$D_{H}=2-H,$$

where the Hurst exponent H can be any real number in the range 0<H<1.

**Figure 1:**
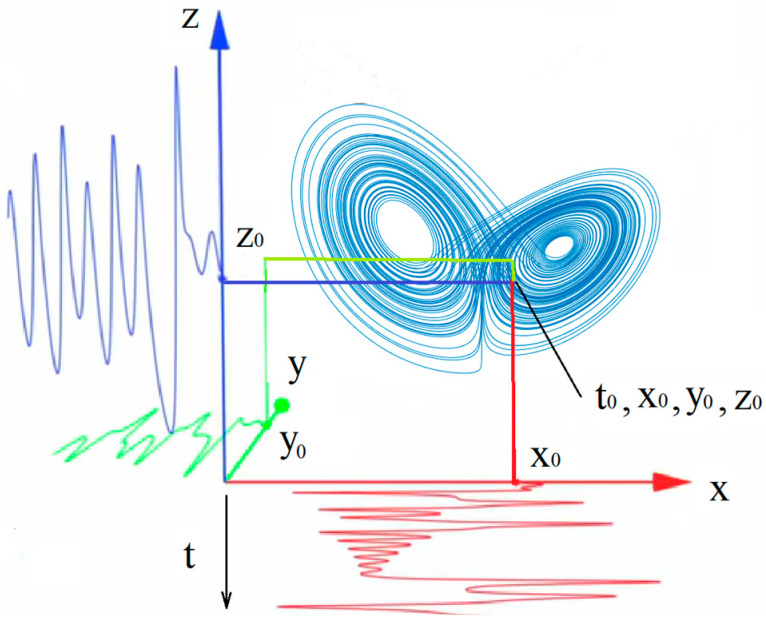
The projections of the Lorenz attractor *x*(*t*) (red), *y*(*t*) (green), *z*(*t*) (blue) on the *x*-subspace, *y*-subspace, and *z*-subspace and a point on the attractor with coordinates ($t_{0}$,$x_{0}$,$y_{0}$,$z_{0}$).

For further reasoning, consider the Lorentz attractor (Figure 1). The introduction of the time axis (*t*) allows us to demonstrate the projections of this attractor on its individual subspaces: the *x*-subspace, *y*-subspace, and *z*-subspace. Accordingly, these projections represent the time series *x*(*t*), *y*(*t*), *z*(*t*). Figure 1 shows the projections of the Lorenz attractor *x*(*t*), *y*(*t*), *z*(*t*) and a point on the attractor with coordinates ($t_{0}$,$x_{0}$,$y_{0}$,$z_{0}$).

Thus, the projections of the attractor on the individual subspaces are time series [30]. We can assume the existence of projections that have behavior similar to the behavior of the fractional Brownian function [29]:

(8) $$B_{H}(t)=\frac{1}{Г\left(H+1/2\right)}{\int_{-\infty }^{t}\left(t-t^{\prime }\right)}^{H-1/2}dB(t^{\prime }),$$

where Г(x) is the Gamma-function, and H is the Hurst exponent. The condition (5) is satisfied: Then

(9) $$H(A_{1})=H(A_{2}),$$

which tells us that projections of the attractor on the individual subspaces have the same Hurst exponent if topological synchronization takes place. If we denote $x_{1}^{*}(t)$ to be a projection on the individual subspace $A_{1}$ and $x_{2}^{*}(t)$ to be a projection on the individual subspace $A_{2}$, then Equation (9) can be written as:

(10) $$H(x_{1}^{*}(t))=H(x_{2}^{*}(t))$$

Equation (10) shows that the topological synchronization condition (5) leads to the interdependence of the fractal properties of the time series. As we demonstrate below, Equation (10) indicates the interdependence between self-similar characteristics of signals on multiple time scales. This fact indicates the fractal synchronization of time series. Using Equations (3)–(10), we now introduce the conditions of fractal synchronization.Fractal synchronization is observed if system (3) generates an attractor $A_{c}$ such that the following apply:

(1) Projections of the attractor on the individual subspaces have behavior similar to the behavior of the fractal Brownian function;
(2) Projections of the attractor on the individual subspaces are dependent on each other, and the strength of their connection is governed by the coupling parameter;
(3) There is an interdependence between self-similar characteristics of signals on multiple time scales.

In Section 2, we give a definition of fractal synchronization, which is based on these conditions.

It is known that an attractor can be detected in any coupled system; this is due to the fact that the value of one variable in some manner limits the eventual values of other variables [31]. Since the brain can be regarded as a nested network of coupled dynamical systems [32], these systems are characterized by attractors. Given the fact that attractor states are generated by the inner framework of a neural network, a system with a large number of neurons may contain different types of attractors, depending on which subsets of cells are more active in the analyzed time period [31]. Models of neural networks characterized by different attractors are widely represented in neuroscience [33,34]. Thus, the presented approach for estimating synchronization, which is based on the analysis of the coupling of attractor projections, can be widely used in the study of stochastic dynamics in the brain.

Since brain functioning demonstrates synchronization of spatiotemporal structures on multiple scales [35], this definition of fractal synchronization can be used to assess the aggregate degree of synchronization between brain regions at multiple time scales. A very specific application in this general framework is filtering, which was presented by Ombao and Pinto [12]. In that work, the “projected” components are exactly the linear band-pass filtered signals, and the interdependence between these projections can be captured linearly through coherency or coherence and also non-linearly through the phase-amplitude coupling. Here, we propose a new method that can be applied in practice to estimate fractal synchronization in real biological systems, in particular, to estimate fractal synchronization between populations of brain neurons.

The self-similar structure of the brain [36,37] indicates the predominance of fractals at all levels of the nervous system [38]. Bieberich [39] suggested that the self-similarity of dendritic branching is necessary for economical information compression and recursive algorithms. There are many works where the authors demonstrate the fractal structure of EEG and fMRI signals [40,41]. Analyzing various studies on the fractal structure of the nervous system in detail, Werner [38] came to the conclusion that the fractal structure is the most economical principle of interaction in the complex dynamics of the nervous system. It allows the nervous system to most optimally interact between different parts of the system at different scales for a coordinated response to external influences.

**Figure 2:**
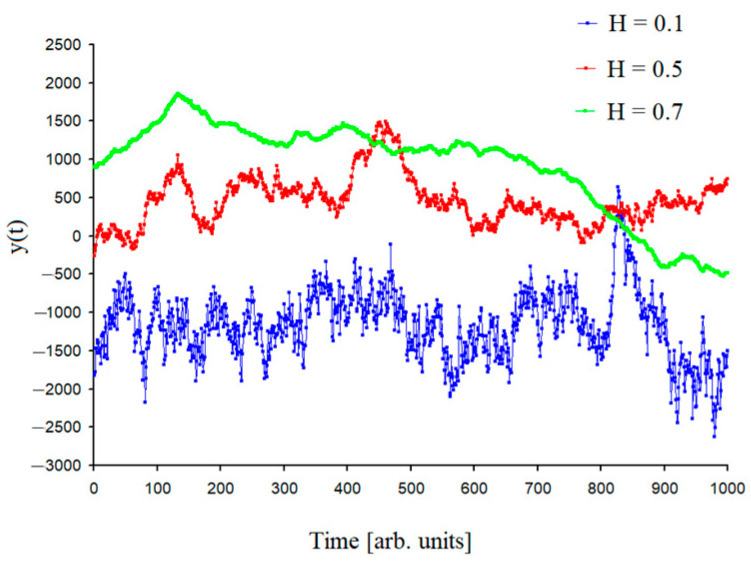
The time series generated by the random midpoint displacement algorithm for the Hurst exponents *H* = 0.1, *H* = 0.5, *H* = 0.7.

The condition of a self-similar signal structure is defined below by means of Equation (11). One of the indicators of self-similarity is the Hurst exponent. It is worth noting that if the value of the Hurst exponent lies in the interval (0.50, 1.00), then such time series will have persistent behavior and characterize long memory processes. If the value of the Hurst exponent lies in the interval (0.00, 0.50), then this is inherent in the mean reverting time series, which are characterized by antipersistent behavior. The Hurst exponent of 0.50 corresponds to Brownian motion [29]. Figure 2 shows the time series generated by the random midpoint displacement algorithm [42] for different Hurst exponents. This example presents a time series with antipersistent behavior H = 0.1, with persistent behavior H = 0.7 and Brownian motion H = 0.5.

**Figure 3:**
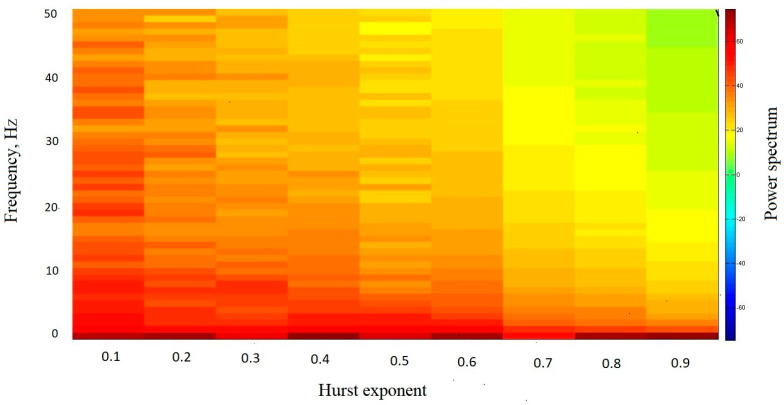
The dependence of the power spectrum on the Hurst exponent for synthetic time series. The corresponding time series were generated using the random midpoint displacement algorithm for different Hurst exponents with a step of 0.1.

It is known that time series, characterized by different Hurst exponents, have different frequency characteristics [29]. Consider how the power spectrum of the time series changes depending on the Hurst exponent. Figure 3 shows the dependence of the power spectrum on the Hurst exponent for synthetic time series. The corresponding time series were generated using the random midpoint displacement algorithm for different Hurst exponents with a step of 0.1.

Figure 3 demonstrates that in the time series characterized by high values of the Hurst exponent (i.e., close to one), low-frequency power predominates. As the Hurst exponent decreases, the contribution of medium-frequency and low-frequency power increases. Thus, the time series with antipersistent behavior is characterized by the predominance of high-frequency components in comparison with the time series with persistent behavior, which is characterized by the predominance of low-frequency components. Thus, the fractal characteristics of a time series are related to its frequency characteristics.

Recently, fractal analysis has been successfully used to analyze EEG signals detecting epileptic seizures. Geng et al. [43] demonstrated the existence of a statistically significant difference (*p* < 0.05) between the Hurst exponents of epileptic EEG signals and interictal EEG signals. Using fractal analysis, Li et al. [44] offered two methods for differentiating epileptiform discharges. Analyzing functional magnetic resonance imaging, which can be modeled as a sum of scale-free EEG “microstates”, Churchill et al. [45] demonstrated that decreases in the Hurst exponent (*H*) were related to three different sources of cognitive effort/task engagement: (1) task difficulty, (2) task novelty, and (3) aging effects. Similarly, decreases in the Hurst exponent were observed in anxiety and fatigue. These results demonstrate the utility of fractal analysis for studying EEG signals, which motivates us to investigate the potential of fractal synchronization for studying EEG signals, particularly during an epileptic seizure. Fractal synchronization will make it possible to estimate the aggregate interdependence between neuronal populations on multiple time scales, which may provide additional information on the development of epileptic seizure dynamics and identifying EEG data. In the following, we describe the most commonly used methods for assessing the interdependence between neuronal populations.

One widely used method to qualify the interdependence between neuronal populations is coherence assessment. However, it is believed that coherency is capable of determining only linear relationships between time series [20]. Thus, this method may not be effective for evaluating non-linear relationships between the underlying dynamical systems. At the same time, fractal synchronization allows us to estimate a non-linear relationship, which is demonstrated in the example of two interacting chaotic systems (Equation (18)). The application of the well-known phase synchronization method makes it possible to evaluate the interdependence between neuronal populations in terms of time-dependent *n*:*m* phase locking, which makes it possible to determine a more general type of interaction between dynamic systems than the coherence. Nevertheless, it is reasonable to use this method when the analyzed signals are approximately oscillatory [20]. Stam et al. [20] proposed a synchronization likelihood measure based on the idea of generalized synchronization to evaluate the interdependence between time series. Stam et al. [20] demonstrated that the synchronization likelihood is able to define the increase in coupling during an epileptic seizure. However, this method does not determine desynchronization, which is characteristic of the beginning of an epileptic seizure [2,3,9,10,11]. In [11,46], methods based on equal-time correlation and correlation matrix analysis were used to evaluate synchronization between neuronal populations during an epileptic seizure. This approach demonstrates the presence of desynchronization at the beginning of an epileptic seizure and an increase in synchronization at the end of an epileptic seizure. This is consistent with the other results [1,2,3,9,10,11].However, Schindler et al. [11] noted that one shortcoming of this method is that it is not frequency selective and may not detect non-linear correlations

Thus, given the shortcomings of the synchronization estimation methods described above, the purpose of this paper is to develop a method for estimating fractal synchronization that would have the following properties:

(1) This method should be effective for evaluating non-linear relationships between the underlying dynamical systems. As we demonstrate below, if system (3) consists of two interacting chaotic systems (non-linear dynamical systems), they will also exhibit fractal synchronization. Thus, fractal synchronization can define a non-linear relationship since the underlying dynamics of such a system are not linear. An important property of fractal synchronization, which allows us to estimate a non-linear relationship, is that the corresponding time series need not be similar to each other.
(2) The proposed method should correctly assess the dynamics of synchronization during an epileptic seizure. Since the brain dynamically unites, synchronizes, and reacts to external and internal stimuli across multiple time scales [7], evaluating the aggregate interdependence between populations of neurons at multiple time scales can provide a more accurate assessment of the dynamics of synchronization during an epileptic seizure.
(3) The results of fractal synchronization estimations should be understandable for physiological interpretation. One of the advantages ofthe interpretation of the proposed method is that this method is based on fractal analysis. There are quite a lot of works related to the physiological interpretation of fractal analysis results.

The disadvantage of this method is that fractal synchronization estimates the interdependence between signals, with behavior similar to the behavior of the fractional Brownian function (8). Accordingly, if the chaotic attractor does not have a fractal dimension, this method is not applicable. However, we believe that this approach can be extended to the more general case. For this purpose, in the future, we plan to develop this idea for the estimation of synchronization on the basis of wavelet analysis.

## 2. Evaluation of Fractal Synchronization

It is known that signals generated by cognitive activity have a fractal structure, i.e., brain cognitive signals display self-similar behavior on various time scales [47]. One hypothesis is that the fractal structure is a consequence of coupling among interdependent processes [48,49]. Chen et al. [49] demonstrated coupling among brain rhythms: δ (0.5–4.0 Hz), θ (4.0–8.0 Hz), α (8.0–12.0 Hz), β (12.0–30.0 Hz), γ (30.0–50.0 Hz). Thus, this coupling covers a wide range of timescales (0.5–50.0 Hz). In order to assess the aggregate interdependence between EEG signals, it is necessary to take into account the coupling on different timescales. One way to study this type of aggregate interdependence is through fractal synchronization.

Consider a stochastic time series {X(t)}, whose behavior is described by the fractal Brownian function (6). According to the definition given in [50], a time series has a fractal structure if the self-similarity condition is satisfied, namely

(11) $$X(\Delta t\times t)\sim \Delta t^{H}\times X(t)$$

for each Δt>0 and t≥0, where H is the Hurst exponent [29,51,52,53,54] and ~ means that the statistical properties of both sides of the equation are identical (two stochastic processes have the same distribution). The scaling properties of this time series can be defined by the Hurst exponent using the generalized variance $\langle \sigma^{2}(\Delta t)\rangle$ [29,51],

(12) $$\langle \sigma^{2}(\Delta t)\rangle \propto \langle {\left[X(t+\Delta t)-X(t)\right]}^{2}\rangle \propto \Delta t^{2H}$$

Thus, the generalized variance $\langle \sigma^{2}(\Delta t)\rangle$ of the fractal time series is a parameter that demonstrates self-similarity on different time scales Δt, which allows using the generalized variance $\langle \sigma^{2}(\Delta t)\rangle$ to estimate the Hurst exponent [29,51,52]. Further, this self-similarity property, which the generalized variance $\langle \sigma^{2}(\Delta t)\rangle$ manifests at different time scales Δt, is used to estimate fractal synchronization.

Let the stochastic time series $X_{1}^{*}(t)$ and $X_{2}^{*}(t)$ satisfying condition (10) and be projections of the attractor of system (3) on the individual subspaces $A_{1}$ and $A_{2}$, respectively. Suppose that the fractal synchronization condition (10) for the projections on the individual subspaces $X_{1}^{*}(t)$ and $X_{2}^{*}(t)$ is satisfied. Then, taking into account Equation (12), we have the following result:

(13) $$\langle \sigma_{1}^{2}(\Delta t)\rangle \propto \langle \sigma_{2}^{2}(\Delta t)\rangle ,$$

where $\langle \sigma_{1}^{2}(\Delta t)\rangle$ and $\langle \sigma_{2}^{2}(\Delta t)\rangle$ are generalized variances of the projections on the individual subspaces $X_{1}^{*}(t)$ and $X_{2}^{*}(t)$. Equation (13) indicates the interdependence between self-similar characteristics of signals $\langle \sigma_{1}^{2}(\Delta t)\rangle$ and $\langle \sigma_{2}^{2}(\Delta t)\rangle$ on multiple time scales. This implies the existence of a functional relation θ between generalized variances on multiple time scales:

(14) $$\langle \sigma_{1}^{2}(\Delta t)\rangle =\theta \left(\langle \sigma_{2}^{2}(\Delta t)\rangle \right)$$

Thus, fractal synchronization (*FS*) is defined as the aggregate strength of a functional relation θ between generalized variances $\langle \sigma^{2}(\Delta t)\rangle$ for two fractal time series, which are projections of an attractor at multiple time scales. In contrast to this, fractal desynchronization is defined as the absence of a functional relationship θ between variances $\langle \sigma^{2}(\Delta t)\rangle$. It should be expected that fractal desynchronization will be characterized by values close to zero. However, it is necessary to take into account the bias, which gives more inflated desynchronization results. In Section 3, we demonstrate the practical evaluation of the desynchronization threshold using the estimation of the significance.

The functional relation (14) can be estimated using mutual information between the values $\langle \sigma_{1}^{2}(\Delta t)\rangle$ and $\langle \sigma_{2}^{2}(\Delta t)\rangle$ on the interval Δt∈ ($\Delta t_{\min};\Delta t_{\max}$), where the values $\Delta t_{\min}$ and $\Delta t_{\max}$ are determined from the condition described below. Applying mutual information will allow for the revealing of a non-linear relationship between the values $\langle \sigma_{1}^{2}(\Delta t)\rangle$ and $\langle \sigma_{2}^{2}(\Delta t)\rangle$ at different time scales in which the parameter Δt varies.

The first step to estimating *FS* is to determine the generalized variances in the trajectories $X_{1}^{*}(t)$ and $X_{2}^{*}(t)$, which are the projections of the attractor $A_{c}$ on the individual subspaces. There are various methods for estimating the generalized variance $\langle \sigma^{2}(\Delta t)\rangle$. The most common are stabilogram diffusion analysis (SDA), detrending moving average (DMA), and detrended fluctuation analysis (DFA) [51,52]. In our work, we used the SDA method, which is described in detail in [52] and, in our opinion, is the simplest and most convenient in the implementation of the above approach since it allows calculating the values of the generalized variance at a minimum value of $\Delta t_{\min}$ = 2, in contrast to the DFA method, where it is necessary to have higher $\Delta t_{\min}$ values to construct an approximating straight line [52,55]. Note that the DMA method has its own potential and will be considered in future work. A comparative analysis of SDA and DMA methods was performed in [52].

Here, the general variance $\langle \sigma^{2}(\Delta t)\rangle$ is estimated as follows:

(15) $$\langle \sigma^{2}(\Delta t)\rangle =\frac{1}{\left(n-1)(N/n\right)}\times \sum_{j=1}^{n-1}\sum_{i=1}^{N/n}{\left(X((j\times \Delta t)+i)-X((j-1)\times \Delta t+i)\right)}^{2}$$

where N is the number of time points, $n=2,3,4,\ldots ,n_{\max}$. The parameter Δt is the interval between two time points and is defined as a condition Δt=N/n. The value $n_{\max}$ depends on the maximum size of the fractal domain.In order to minimize the saturation effects due to finite size, $n_{\max}$ must satisfy condition $n_{\max}<<N$ [51]. The square displacement ${\left(X((j\times \Delta t)+i)-X((j-1)\times \Delta t+i)\right)}^{2}$ is calculated for all pairs of points located on the same intervals Δt. The second step is to evaluate mutual information between the values $\langle \sigma_{1}^{2}(\Delta t)\rangle$ and $\langle \sigma_{2}^{2}(\Delta t)\rangle$ on the interval Δt∈ ($\Delta t_{\min};\Delta t_{\max}$), where the values $\Delta t_{\min}$ and $\Delta t_{\max}$ are determined from the above conditions. Computing mutual information uncovers potential non-linear relationships between the values $\langle \sigma_{1}^{2}(\Delta t)\rangle$ and $\langle \sigma_{2}^{2}(\Delta t)\rangle$ at different time scales in which the parameter Δt varies. This allows us to estimate the fractal synchronization according to Equation (14).

For calculating mutual information, a histogram-based approach developed in [56] is used. The method for estimating mutual information [56] between the values $\langle \sigma_{1}^{2}(\Delta t)\rangle$ and $\langle \sigma_{2}^{2}(\Delta t)\rangle$ is described as follows. Consider a system *A* with $M_{A}$ possible states, i.e., *A* takes on one of the following unique values $a_{1},\ldots ,a_{M_{A}}$, each with probability $p(a_{i})$, $i=1,\ldots ,M_{A}$. Analogically, consider a system *B* with $M_{B}$ possible states, i.e., *B* takes on one of the following unique values $b_{1},\ldots ,b_{M_{B}}$, each with probability $p(b_{j})$, $i=1,\ldots ,M_{B}$. Thus, the mutual information between systems *A* and *B* is defined as follows:

(16) $$MI(A,B)=\sum_{i=1}^{M_{A}}\sum_{j=1}^{M_{B}}p(a_{i},b_{j})\log\left(\frac{p(a_{i},b_{j})}{p(a_{i})p(b_{j})}\right)$$

where $p(a_{i},b_{j})$ designates the joint probability that *A* is in the state $a_{i}$ and *B* is in state $b_{j}$. If the measurements of systems *A* and *B* are statistically independent, then $p(a_{i},b_{j})$ = $p(a_{i})$$p(b_{j})$ and thus *MI*(*A*,*B*) = 0. In contrast, when *A* and *B* are statistically dependent, then *MI*(*A*,*B*) > 0. To estimate the joint probability $p(a_{i},b_{j})$ and the marginal probabilities $p(a_{i})$ and $p(b_{j})$, in (16), we use the histogram method described in [56].

Additionally, in our article, the power spectrum of EEG signals was estimated.In this paper, the power spectrum of an EEG signal $X_{t}$ sampled at discrete time points t=1,…,N is estimated using the periodogram, which we define below:

(17) $$S(\omega_{j})=\frac{1}{2\pi N}{\left|\sum_{t=1}^{N}X_{t}e^{-it\omega_{j}}\right|}^{2},$$

with fundamental Fourier frequencies $\omega_{j}=2\pi j/N$ and $j=1,\ldots ,\left[(N-1)/2\right]$, where $\left[.\right]$ denotes the integer part [57]. The largest frequency to be resolved is the Nyquist frequency $f_{Ny}=\frac{\omega_{Ny}}{2\pi }=\frac{1}{2\Delta }$, where Δ is the sampling interval. The periodogram in Equation (17) is asymptotically unbiased for the true spectrum, but it is inconsistent because its variance does not decay to 0 when the length *N* of the time series goes to infinity. Thus, a mean-squared consistent estimator for the spectrum is constructed by smoothing the periodograms. One such smoother is the Hanning taper, which reduces the Gibbs effect resulting from the sharp edges of the regular taper (raw).

## 3. Conceptual Examples

### 3.1. Synchronization Properties of Fractal Synchronization

To demonstrate the synchronization properties of the proposed *FS*, we studied the coupled Lorenz model systems [17] with an increasing coupling parameter. In this setup, we call the system X (with state variable $x_{i}$) the “driving” system, and we denote the response system to be Y (with state variable $y_{i}$). The coupled systems of Lorentz models [17] are described by the following difference equations:

(18) $$\frac{d{\vec{x}}_{1}}{dt}=\sigma (x_{2}-x_{1})\;\frac{d{\vec{x}}_{2}}{dt}=x_{1}(R_{0}-x_{3})-x_{2}\;\frac{d{\vec{x}}_{3}}{dt}=x_{1}x_{2}-bx_{3}\;\frac{d{\vec{y}}_{1}}{dt}=\sigma (y_{2}-y_{1})\;\frac{d{\vec{y}}_{2}}{dt}=y_{1}(R_{1}-y_{3})-y_{2}\;\frac{d{\vec{y}}_{3}}{dt}=y_{1}y_{2}-by_{3}+C(x_{3}-y_{3})$$

where σ=10, $R_{0}=28$, $R_{1}=28.001$, $b=\frac{8}{3}$. For the coupled Lorenz systems (18), we analyzed the *FS* between the third components $x_{3i}$ and $y_{3i}$ according to [17]. To demonstrate the effect of fractal synchronization, the dynamical system Equation (18) was obtained for values of coupling strength *C* increasing from *C* = 0 to *C* = 2.4. We generated time series $x_{i}$ and $y_{i}$ for dynamical systems containing 50,000 points. Further, the generated time series was divided into 50 intervals, each containing 1000 points, at which fractal synchronization was estimated.

**Figure 4:**
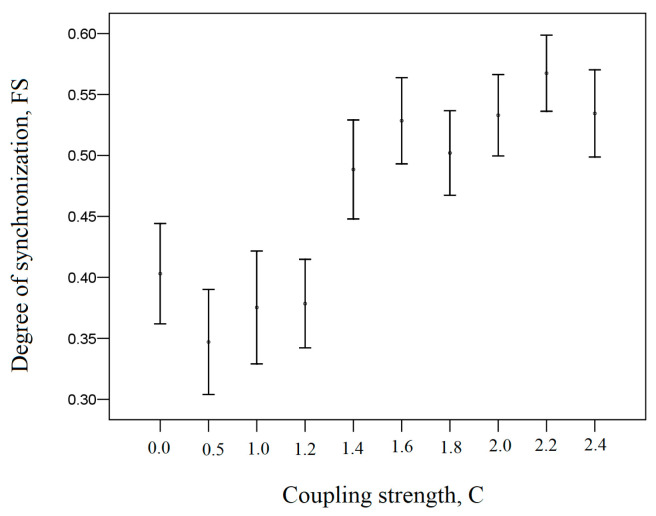
The degree of fractal synchronization *FS* versus the coupling strength *C* for the system (18).

Figure 4 shows the dependence of the degree of fractal synchronization *FS* on coupling strength *C* for the system (18).

Figure 4 demonstrates that fractal synchronization increases with increasing coupling strength. The fractal synchronizations *FS* in interval *C*∈[0.0, 1.2] are statistically significantly different (*p* < 0.05, Dunn’s Test of Multiple Comparisons) and have lower values than the fractal synchronizations *FS* in interval *C*∈[1.4, 2.4]. This is due to the fact that with increasing parameter *C*, the system (Equation (18)) generates an attractor whose projections become more dependent on each other. This leads to reinforcing the functional relation θ (Equation (14)) between the generalized variances considered projections of the attractor $x_{3i}$ and $y_{3i}$ (Equation (18)).

Thus, an increase in fractal synchronization indicates that the analyzed system generates an attractor whose projections become more dependent on each other. This leads to reinforcing the functional relation θ between the generalized variances $\langle \sigma^{2}(\Delta t)\rangle$. As generalized variance $\langle \sigma^{2}(\Delta t)\rangle$ is calculated on different time scales Δt, the increase in fractal synchronization indicates an increase in the functional relation between the two signals on different time scales Δt. It follows that fractal synchronization characterizes the aggregate strength of the relation between generalized variances $\langle \sigma^{2}(\Delta t)\rangle$ for two fractal time series, which are projections of an attractor at multiple time scales. Thus, fractal synchronization can reflect trends associated with the fractal dynamics of the system underlying the process under study.

Nevertheless, as can be seen from Figure 4, the fractal synchronization is not close to zero at small values of coupling strength. This suggests the existence of a bias in the estimation of fractal synchronization

### 3.2. Bias

One of the important procedures for assessing the properties of measurements of interdependence is to estimate the bias. It is assumed that for small values of coupling strength, the fractal synchronization of the two time series is close to the synchronization of the two independent time series. Random shuffling of the time series, which was used in our work to obtain surrogate series [58], removes the original fractal structure of the time series.

Since the proposed calculation method has a bias at small values of the coupling strength, evaluating the significance *S* is used to estimate the bias. To evaluate significance *S*, the (standardized) *FS* Z-score was used (see Equation (19)) [58].

(19) $$S=\frac{FS-\langle {FS}_{shuffle}\rangle }{\sigma_{shuffle}},$$

where $\langle {FS}_{shuffle}\rangle$ is the mean of the shuffled values, and $\sigma_{shuffle}$ is the standard deviation. Here, the standardized fractal synchronization, under the null, has a standard normal distribution (mean 0 and variance 1). Thus, the observed Z-score value of 3 approximately corresponds to the significance level *p*-value = 0.01. Thus, if the significance S ≤ 3, then the fractal synchronization *FS* of the analyzed signals is interpreted asnot having exceeded the fractal synchronization *FS* of the independent shuffled signals (which mimics the properties of the null). Thus, in that case, we conclude that there is no fractal synchronization (or no sufficient evidence for *FS*). This phenomenon is interpreted as fractal desynchronization.

**Figure 5:**
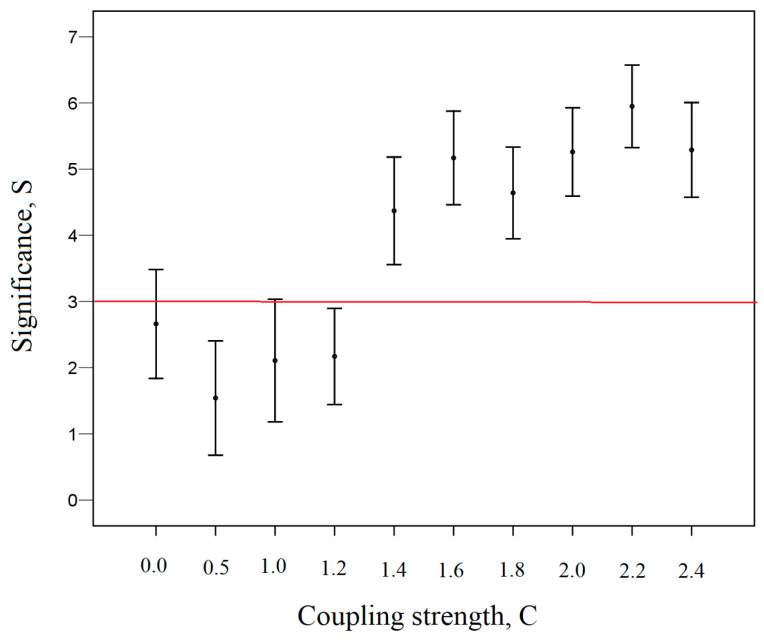
The significance *S* of fractal synchronization *FS* versus the coupling strength *C* for the fractal synchronization *FS*, presented in Figure 4.

Figure 5 demonstrates the change in significance from the strength of coupling *C* for the fractal synchronization *FS* shown in Figure 4. Fractal synchronization *FS* was calculated for the time series $x_{3i}$ and $y_{3i}$ of the Equation (18).

Figure 5 demonstrates that for small coupling strengths (C ≤ 1.2), the fractal synchronization of the analyzed signals is no greater than the fractal synchronization of the independent shuffled signals. Thus, if the significance S ≤ 3, then this indicates the absence of fractal synchronization. In this case, we have fractal desynchronization.

## 4. Analysis of Fractal Synchronization of EEG Signals

### Clinical Datasets

**Figure 6:**
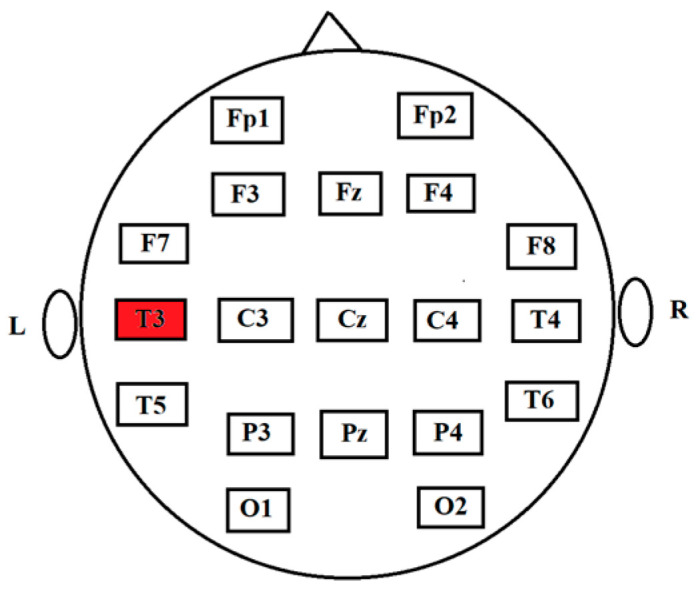
The disposition of the scalp electrodes. The abbreviations designate the disposition on the scalp: frontal polar (Fp), frontal (F), central (C), temporal (T), parietal (P), and occipital (O). Channel T3, which is highlighted in red, is the focus of the epileptic seizure.

In this study, we examined in detail the estimation of fractal synchronization using the EEG data collected at the Epilepsy Center (PI: Dr. Beth Malow) at the University of Michigan from the subject who experienced a spontaneous epileptic seizure. The EEG was sampled at 100 Hz and lasted for about 500 s. There were 50,000 time points in each channel. The electrodes were located according to the 10–20 system. Figure 6 demonstrates the disposition of the scalp electrodes. The T3 channel highlighted in red is the focus of the epileptic seizure. Channel Cz was used as a reference electrode. Artifact Subspace Reconstruction (ASR) technique [59] was used to pre-process the EEG signals in the channels Fp1, Fp2, F3, F4, C3, C4, P3, P4, O1, O2, F7, F8, T3, T4, T5, T6, and Pz.

The approximate time of the onset of the epileptic seizure is ~340 s. A detailed protocol for recording EEG signals and the results of their study are presented in the articles by Ombao et al. [60,61,62,63]. Additionally, some records (subject 13, subject 34, subject 36, subject 78) from the publicly available Neonatal Epileptic EEG dataset from Stevenson et al. [64] were used. The dataset contains preictal and ictal periods measured during sleep. All subjects had recordings with a sample frequency of 256 Hz. Authors [64] used a reference electrode “at midline”, but the authors did not specify the exact location of this electrode. The subjects in question, according to the description of the databases, have the following diagnoses: subject 13—cardiac anomalies; subject 34—severe asphyxia, infarction; subject 36—status post cardiac operation; subject 78—acute ischemia, infarction.

## 5. Results

It is known that at the onset of an epileptic seizure, there is a decrease in long-range synchronization [2]. This allows the brain to reduce the exchange of information between the epilepsy focus and the rest of the neural population [6,32,65,66]. Thus, this localization of information prevents the brain from spreading incorrect information emanating from the focus of epilepsy in order to prevent more significant disturbances in the process of information integration and coordination of brain activity. At the same time, at the onset of an epileptic seizure, there is local synchronization in the focus of an epileptic seizure [2,67], which allows an intense exchange of information between neurons at a short distance (less than 1 mm). Such a process probably allows the formation of new connective chains of neurons [67] in the local space (less than 1 mm), which can eliminate the resulting disorder. It is possible that this process of formation of new neuronal connections involves a population of neurons, which Quyen et al. [65] referred to as the “idle” population of neurons. The greater the distance from the epilepsy focus at the onset of an epileptic seizure, the weaker the synchronization between the population of neurons belonging to the epilepsy focus and the populations of neurons from other brain regions. At the end of an epileptic seizure, namely, after the elimination of neuronal dysfunction in the epilepsy focus, the local synchronization decreases, and the long-range synchronization, on the contrary, increases [2]. As a result, the exchange of information between different parts of the brain, necessary for the process of integrating information and coordinating brain activity, is restored.

In this paper, we investigated how long-range synchronization changes during an epileptic seizure. The long-range synchronization was assessed using fractal synchronization between the epilepsy focus and other neuronal populations. We believe that if the behavior of fractal synchronization corresponds to the above-described behavior of long-range synchronization during an epileptic seizure, then the fractal synchronization method is correct and can be used to assess long-range synchronization during an epileptic seizure for research and medical purposes.

The application of fractal synchronization to EEG signals makes it possible to determine how strongly the generalized variances $\langle \sigma^{2}(\Delta t)\rangle$ of EEG signals are related at different time scales. The stronger the relationship between the generalized variances $\langle \sigma^{2}(\Delta t)\rangle$, the stronger the interdependence between EEG signals. Accordingly, the greater the flow of information between neuronal populations, which are characterized by these EEG signals. Thus, fractal synchronization makes it possible to evaluate the change in the flow of information between neuronal populations. In particular, fractal desynchronization indicates a lack of information exchange.

Considering the above, in this work, we investigated *FS* synchronizations between the EEG signal of channel T3, which is the focus of epilepsy, and other EEG signals of the left and right hemispheres of the brain. Thus, synchronizations between the T3 channel and left hemisphere brain channels were evaluated: T3-Fp1, T3-F3, T3-C3, T3-F7, T3-T5, T3-P3, T3-O1. Similarly, synchronizations between the T3 channel and the right brain hemisphere channels were evaluated: T3-Fp2, T3-F4, T3-F8, T3-T4, T3-C4, T3-T6, T3-P4, T3-O2. In this study, the width of the window used to calculate the corresponding values (fractal synchronization, Hurst exponent) is 1000 points.

**Figure 7:**
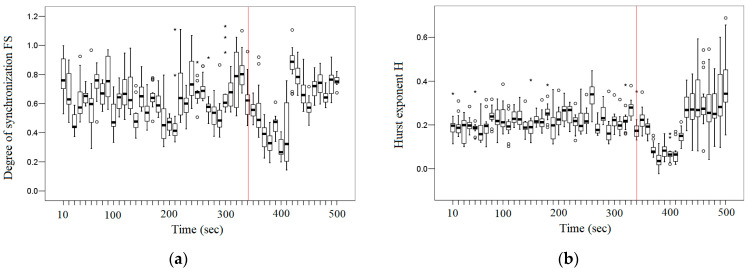
(**a**) The dynamic change in the average *FS* between the EEG signal of the T3 channel and the EEG signals of the right and left hemisphere brain channels during the epileptic seizure. (**b**) The dynamic change in the average *H* of the EEG signals of the right and left hemisphere brain channels during the epileptic seizure.

To identify general patterns (tendency), we consider the dynamic change in the average *FS* between the EEG signal of the T3 channel and the EEG signals of the right and left brain hemispheres. Figure 7a demonstrates the dynamic change in the average *FS* between the EEG signal of the T3 channel and the EEG signals of the right and left hemisphere brain channels. The vertical red line indicates the approximate time of the onset of the epileptic seizure. Figure 7b shows the change in Hurst exponent (*H*) for the EEG signals of the corresponding channels. On the boxplots shown here, outliers are identified;note the different markers for “out” values (small circle) and “far out” (marked with a star).

Figure 7a,b demonstrate that there is a general trend in synchronization and fractal behavior for the studied pairs. Unfortunately, visually, we cannot determine the onset of an epileptic seizure. However, knowing that the approximate time of the onset of the epileptic seizure is ~340 s, we can distinguish the pre-seizure period (~10–340 s). Further, visually, we can highlight the initiation period (~350–410 s), which is characterized by low fractal synchronization values, and the termination period (~420–500 s), which is characterized by higher values of fractal synchronization than the initiation period. Small values of fractal synchronization at the initiation period may indicate fractal desynchronization.

**Figure 8:**
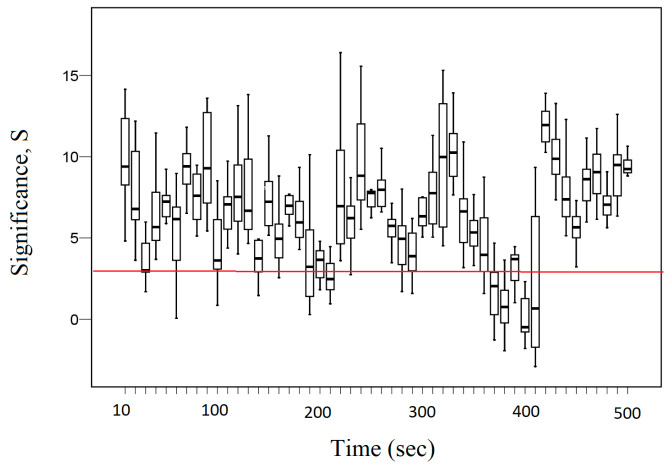
Dynamic change in the average significance S between the EEG signal of the T3 channel and the EEG signals of the right and left hemisphere brain channels.

In order to determine the interval at which fractal desynchronization occurs, consider in Figure 8 the dynamic change in the average significance *S* (Equation (19)) between the EEG signal of the T3 channel and the EEG signals of the right and left hemisphere brain channels. The red line indicates the boundaries of fractal desynchronization at *S* = 3 (the significance level *p* = 0.01 approximately corresponds to the Z-scores = 3).

Figure 8 demonstrates that in the initial period in an interval of 370–410 s, we can quite clearly observe the effect of fractal desynchronization, as the average significance *S* ≤ 3 in this interval. Thus, during the initiation period (~350–410 s), there is a period of fractal desynchronization (~370–410 s). As we can see, the period of fractal desynchronization is followed by a termination period (~420–500 s). Thus, the start and end points of fractal desynchronization were determined at the significance level *p* = 0.01, to which the Z-scores = 3 corresponds.

One of the main limitations of the proposed tool, in its current form, is that of the interpretation of brain functional connectivity. This limitation is inherent in the nature of the EEG (sensor) signals, which are not spatially localized due to the volume conduction. Thus, in the current form, when a pair of EEG signals exhibit a statistically significant fractional synchronization, we cannot interpret this as the synchronization of the subpopulation of neurons on the cortex. Our interpretation is limited to that at the sensor level (rather than the source level). Rather, we only state that fractal desynchronization is a reduced amount of information transfer between the two EEG sensors. Moreover, there are potential tools for reducing the spurious effects due to volume conductance [68,69]. This will be the subject of our future investigation, and this has to be rigorously conducted with several experiments.

Note, however, that despite the limitations of the interpretation, *FS* can still be considered as a viable “feature” in EEG signals for the purpose of classification and discrimination. Moreover, for more general signals (without the practical problem of volume conduction), the proposed *FS* can be used as a potential measure of dependence in a network of time series in the same way as other measures of dependence such as cross-correlation, partial cross-correlation, coherence, partial coherence, and partial directed coherence.

To identify features for the selected periods, we perform a statistical comparison of the fractal synchronization *FS* for the pre-seizure period $FS_{P}$, fractal desynchronization $FS_{D}$, and termination period $FS_{T}$. Since the empirical distribution of the estimated values differs from a normal distribution (*p*-value < 0.05, Kolmogorov–Smirnov test), we proceeded to use the median (*Me*) in order to evaluate the central values of *FS* and applied the first quartile $Q_{1}$ and third quartile $Q_{3}$ to evaluate the variation or spread of the distribution of the considered values. Table 1 shows the results achieved for the fractal synchronization *FS* for the pre-seizure period $FS_{P}$, fractal desynchronization $FS_{D}$, and termination period $FS_{T}$.

The Kruskal–Wallis rank sum test revealed a statistically significant difference (*p* < 0.01) between $FS_{P}$, $FS_{D}$, and $FS_{T}$. Accordingly, fractal desynchronization $FS_{D}$ has lower values (*p*-value < 0.01, Dunn’s Test of Multiple Comparisons) compared to the fractal synchronization of the pre-seizure period $FS_{P}$. The termination period $FS_{T}$ is characterized by higher values (*p*-value < 0.01, Dunn’s Test of Multiple Comparisons) than the fractal synchronization of the pre-seizure period $FS_{P}$. Thus, the fractal desynchronization period is characterized by the weakest dependence on different time scales between the generalized variance $\langle \sigma^{2}(\Delta t)\rangle$ of the EEG signal of the T3 channel and the generalized variances $\langle \sigma^{2}(\Delta t)\rangle$ of the EEG signals of the right and left hemisphere brain channels.

In contrast, the termination period is characterized by the strongest dependence on different time scales between the generalized variance $\langle \sigma^{2}(\Delta t)\rangle$ of the EEG signal of the T3 channel and the generalized variances $\langle \sigma^{2}(\Delta t)\rangle$ of the EEG signals of the right and left hemisphere brain channels. However, further studies of fractal synchronization that were performed during an epileptic seizure with other EEG recordings did not reveal an increase in fractal synchronization during the termination period $FS_{T}$ compared to the pre-seizure period $FS_{P}$. It is possible that this effect is a special case. It is worth noting that when analyzing other records, the results of which are given below, we did not know the focus of epilepsy. It is possible that this discrepancy is related to this. However, we do not ignore this result; perhaps this is one of the mechanisms for stopping an epileptic seizure.

**Figure 9:**
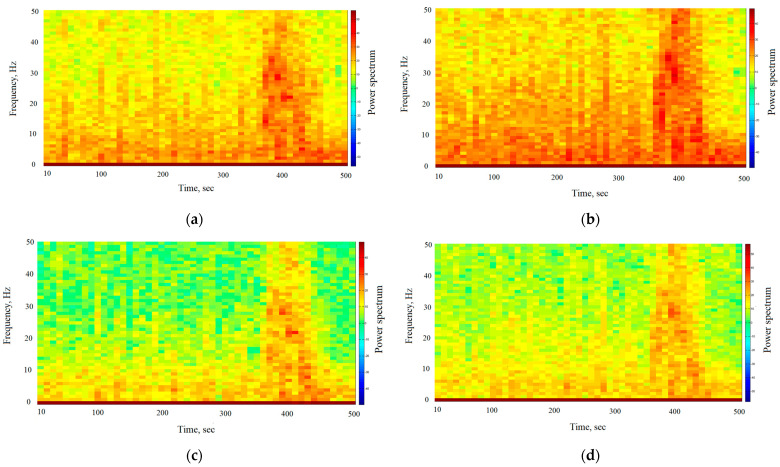
The power spectrum during the epileptic seizure for channels (**a**) T3, (**b**) T4, (**c**) C3, (**d**) C4.

Solomon et al. [70] establish that decreases in synchrony accompany increases in high-frequency power and that this fundamental relationship between power and synchrony manifests itself throughout the human brain. Thus, we can expect that the decrease in fractal synchronization should be accompanied by an increase in high-frequency power. We consider the frequency characteristics for the T3, T4, C3, C4 channels during the epileptic seizure in more detail. Figure 9 shows the power spectrum for the T3, T4, C3, C4 channels during the epileptic seizure. The power spectrum was estimated according to Equation (18). The width of the window for calculating the power spectrum of EEG signals is 1000 points.

By analyzing Figure 9a–d, we can see that in the pre-seizure period, in all the EEG channels, power is concentrated at the lower frequencies (delta band, theta band, alpha band: 0.5–12 Hz). The fractal desynchronization interval (370–410 s) is characterized by an increase in the power of medium (beta band: 13–30 Hz) and high frequencies (gamma band: 30–50 Hz). The corresponding frequency range (15–40 Hz), which is observed during desynchronization at the onset of an epileptic seizure, was described in [3]. The highest powers are highlighted at 20–30 Hz in all the EEG channels. It should be mentioned that de Curtis et al. [71] support the idea that synchronization through inhibition is crucial for the generation of fast activity at 20–30 Hz observed at the start of a focal seizure. Thus, we see that fractal desynchronization is accompanied by an increase in high-frequency power, which coincides with the results in [70]. The termination period (420–500 s) demonstrates a predominance of low-frequency power (delta band, theta band, alpha band: 0.5–12 Hz) in all the EEG channels. The predominance of low-frequency power with increasing synchronization at the end of an epileptic seizure has been described in [71]. Low-frequency connections support information integration or coordinated brain activity [70].

For further analysis of the fractal dynamics, consider the change in the Hurst exponent at the selected periods. Table 2 shows the results obtained for the Hurst exponent (*H*) for the pre-seizure period $H_{P}$, for the fractal desynchronization period $H_{D}$, and for the termination period $H_{T}$.

The Kruskal–Wallis test revealed a statistically significant difference (*p* < 0.01) between $H_{P}$, $H_{D}$, and $H_{T}$. Accordingly, the Hurst exponent of the fractal desynchronization period $H_{D}$ has lower values (*p*-value < 0.01, Dunn’s Test of Multiple Comparisons) compared to the Hurst exponent of the pre-seizure period $H_{P}$. The Hurst exponent of the termination period $H_{T}$ is characterized by higher values (*p*-value < 0.01, Dunn’s Test of Multiple Comparisons) than the Hurst exponent of the pre-seizure period $H_{P}$. As shown in [45], a decrease in the Hurst exponent indicates an increase in brain effort, which puts the brain in a state of more limited dynamic range. Thus, the lower values of the Hurst exponent of the fractal desynchronization period $H_{D}$ compared to the Hurst exponent of the pre-seizure period $H_{P}$ indicates a more limited dynamic range of the brain on the fractal desynchronization period.

Additionally, some records from the Neonatal Epileptic EEG dataset from Stevenson et al. [64] were used to evaluate fractal synchronization. Since the focus of epilepsy is not indicated in the description of the database, the calculation of fractal synchronization was performed between various channels of the right and left hemispheres of the brain: Fp1-Fp2, Fp2-F3, F3-F4, F4-C3, C3-C4, C4-P3, P3-P4, P4-O1, O1-O2, O2-F7, F7-F8, F8-T3, T3-T4, T4-T5, T5-T6. ASR technique [59] was used to pre-process the EEG signals in the channels.

**Figure 10:**
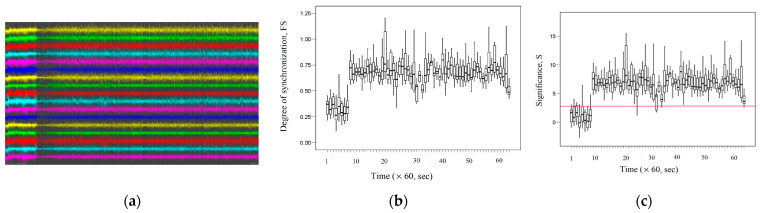
Subject 34: seizure interval: (0–451) seconds. (**a**) The records of EEG signals in the considered channels during the interval (0, 3600) seconds; (**b**) dynamic change in the average *FS* between the EEG signals of the considered channels; (**c**) dynamic change in the average significance *S* between the EEG signals of the considered channels.

Figure 10a shows a graph of the recorded EEG signals in the channels listed above for the first 3600 s for subject 34. According to the information provided in the database description [64], the epileptic seizure for subject 34 is observed in the interval (0–451) seconds. Figure 10b shows the dynamic change in the average *FS* between the EEG signals of the channels listed above during the first 3600 s. Figure 10c shows the dynamic change in the average significance *S* between the EEG signals of the considered channels during the first 3600 s.

As we can see, Figure 10c demonstrates the presence of fractal desynchronization in the interval (0–451) seconds, as the average significance *S* ≤ 3. The average value of *FS* = 0.33 (0.26–0.40) during the epileptic seizure is statistically significantly different (*p* < 0.05) and has a smaller value compared to the average value of *FS* = 0.68 (0.61–0.76) for post-epileptic seizure interval during (1295–3600) seconds. Thus, the termination of the epileptic seizure is accompanied by increased fractal synchronization.

**Figure 11:**
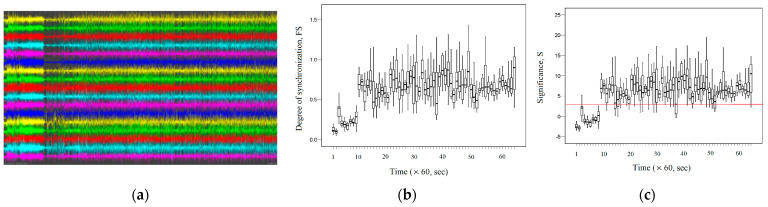
Subject 36: seizure interval 1: (0–109) seconds; seizure interval 2: (187–528) seconds.(**a**) The records of EEG signals in the considered channels during the interval (0, 3600) seconds; (**b**) the dynamic change in the average *FS* between the EEG signals of the considered channels; (**c**) dynamic change in the average significance *S* between the EEG signals of the considered channels.

Figure 11a demonstrates the EEG signal recordings for subject 36 for the first 3600 s. According to the information provided in the database description, epileptic seizures for subject 36 are observed at intervals of (0–109) seconds and (187–528) seconds. Respectively, Figure 11b shows the change in fractal synchronization during an epileptic seizure, and Figure 11c shows the change in significance for fractal synchronization.

Figure 11c demonstrates the presence of fractal desynchronization during the epileptic seizure (*S* ≤ 3 in this interval). Figure 11b shows that during the (0–528) seconds, the average value of *FS* = 0.20 (0.13–0.26) is statistically significantly different (*p* < 0.05) and has lower values compared to the average value of *FS* = 0.66 (0.56–0.81) after the epileptic seizure during (1295–3600) seconds. As in the previous example, the termination of the epileptic seizure is characterized by an increase in fractal synchronization.

**Figure 12:**
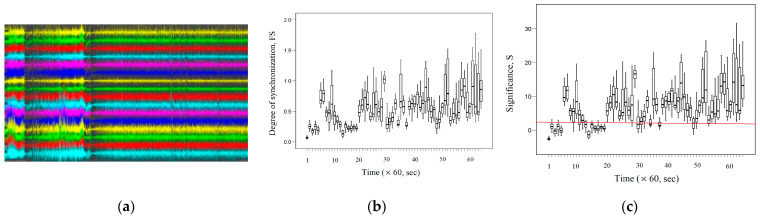
Subject 13: seizure interval 1: (0–291) seconds; seizure interval 2: (799–1294) seconds.(**a**) The records of EEG signals in the considered channels during the interval (0, 3600) seconds; (**b**) the dynamic change in the average *FS* between the EEG signals of the considered channels; (**c**) the dynamic change in the average significance *S* between the EEG signals of the considered channels.

Figure 12a shows recordings of EEG signals for subject 13 for the first 3600 s, during which two epileptic seizures were detected. The first epileptic seizure occurred in the interval (0–291) seconds, and the second epileptic seizure occurred 508 s later in the interval (799–1294) seconds. Figure 12b demonstrates the dynamic change in fractal synchronization *FS* during the first and second epileptic seizures. Figure 12c demonstrates the dynamic change in significance *S* for fractal synchronization during epileptic seizures.

Figure 12c demonstrates fractal desynchronization (*S* ≤ 3) at time intervals that correspond to the time intervals of epileptic seizures. Figure 12b demonstrates a decrease in fractal synchronization at two time intervals that correspond to the time intervals at which epileptic seizures occurred. Thus, during (0–291) seconds, the average value of *FS =* 0.20 (0.14–0.25) is statistically significantly different (*p* < 0.05) and has lower values compared to the average value of *FS* = 0.52 (0.40–0.69) after epileptic seizure during (292–798) seconds. And during (799–1294) seconds, the average value of *FS =* 0.25 (0.20–0.34) is statistically significantly different (*p* < 0.05) and has lower values compared to the average value of *FS* = 0.57 (0.41–0.78) after epileptic seizure during (1295–3600) seconds.

**Figure 13:**
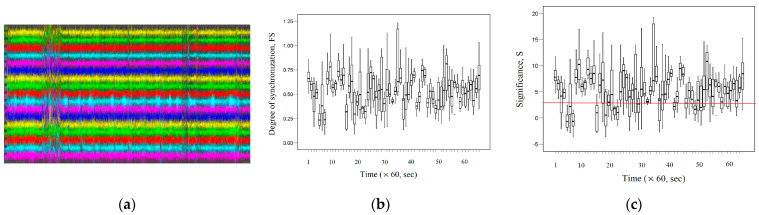
Subject 78: seizure interval: (300–420) seconds;seizure interval: (840–900) seconds; seizure interval: (2220–2280) seconds; seizure interval: (2520–2580) seconds.(**a**) The records of EEG signals in the considered channels during the interval (0, 3600) seconds; (**b**) the dynamic change in the average *FS* between the EEG signals of the considered channels; (**c**) the dynamic change in the average significance *S* between the EEG signals of the considered channels.

Figure 12c demonstrates fractal desynchronization (*S* ≤ 3) at time intervals that correspond to the time intervals of epileptic seizures. Figure 12b shows that in both cases, the termination of epileptic seizure is accompanied by an increase in fractal synchronization. Figure 13a shows the EEG signal recordings for subject 78 for the first 3600 s. According to the description of these signals, epileptic seizures are observed at short time intervals during the first 3600 s. Figure 13b demonstrates the dynamic changes in fractal synchronization during epileptic seizures. Figure 13c shows the dynamic change in significance for fractal synchronization.

Figure 13c shows that fractal desynchronization (*S* ≤ 3) is observed at intervals that are characterized by the presence of an epileptic seizure. Figure 13b demonstrates a decrease in fractal synchronization values at intervals corresponding to epileptic seizures. Thus, during (300–420) seconds, the average value of *FS* = 0.27 (0.21–0.32); during (840–900) seconds, the average value of *FS* = 0.32 (0.26–0.35); during (2220–2280) seconds, the average value of *FS* = 0.19 (0.14–0.26); during (2520–2580) seconds, the average value of *FS* = 0.16 (0.14–0.20) are statistically significantly different (*p* < 0.05) and have lower values compared to the average value of *FS* = 0.44 (0.30–0.66) during the absence of an epileptic seizure. However, subject 78’s recordings have seizure intervals that are not characterized by desynchronization. This indicates the heterogeneity of the nature of epileptic seizures.

Thus, the results shown in Figure 10, Figure 11, Figure 12 and Figure 13 demonstrate a decrease in fractal synchronization and the presence of fractal desynchronization during epileptic seizure, which is consistent with the above results.

## 6. Discussion

We proposed a new method for analyzing and evaluating non-linear coupling between two time series. For the theoretical basis of this method, the concept of fractal synchronization, based on the concept of stochastic synchronization, was introduced. Our goal for this paper is to present the viability of the proposed tool for assessing dependence through fractal synchronization. As is known, the minimum demand for any synchronization criterion varies in a systematical manner when the coupling strength between two dynamical systems enlarges [20]. To assess fractal synchronization, we used the coupled Lorenz model systems (18) with the coupling parameter *C*. These dynamical systems are connected in such a way that with an increase in the value of *C*, the coupling strength between the systems increases. As a result of increasing the coupling strength, the system generates an attractor whose projections become more dependent on each other. This strengthens the functional relation θ between the generalized variances $\langle \sigma^{2}(\Delta t)\rangle$ of the attractor projections on time scales Δt. Fractal synchronization allows for estimating the aggregate strength of the connection on multiple time scales between generalized variances $\langle \sigma^{2}(\Delta t)\rangle$ of two projections of the attractor, which are time series with a fractal structure.

Since the proposed calculation method has a bias at small values of the coupling strength, evaluating the significance of *S* is used to estimate the bias. To evaluate the significance, the Z-score was used. We assume that fractal synchronization *FS* has the Gaussian distribution. Then, the parameter Z-score = 3 approximately corresponds to the significance level *p* = 0.01. Thus, if the significanceis S ≤ 3, then the fractal synchronization *FS* of the analyzed signals does not exceed the fractal synchronization *FS* of the independent shuffled signals. Thus, in this case, there is no fractal synchronization. We denote this effect as fractal desynchronization.

The main task of this current paper is to propose a data analytic tool for assessing fractal synchronization in EEGs. It was demonstrated that this method can be used to assess the interdependence of EEG signals during an epileptic seizure since the obtained result is consistent with the results of other authors. In this paper, we discussed the novel findings that have not been reported in previous analyses of this Michigan seizure dataset. Additionally, some records from the publicly available Neonatal Epileptic EEG dataset from Stevenson et al. [64] were used to evaluate fractal synchronization.

As a result of EEG studies recorded during an epileptic seizure, this paper demonstrated that fractal synchronization *FS* effectively determines changes in the interdependence between EEG signals. The current work does not highlight the onset of an epileptic seizure. However, fractal synchronization demonstrates the presence of desynchronization during an epileptic seizure. This result is consistent with the results obtained by other authors. So in the work [72], the authors claim that “studies on network synchronization and on the networks’ synchronizability indicate that the changing network topology during seizures is accompanied by initially decreased network synchronization and decreased stability of the globally synchronized state, both of which increase already prior to seizure end. These synchronization phenomena may thus be considered as an emergent (network-topology-mediated) self-regulatory mechanism for seizure termination”.

However, using the estimate of significance (Equation (19)) [58], the fractal synchronization method allows one to highlight the beginning and end of fractal desynchronization. Fractal desynchronization indicates a decrease in the information exchange associated with fractal dynamics between the EEG sensor of the epilepsy focus and other EEG sensors. At the same time, analysis of the fractal dynamics, using the Hurst exponent, revealed a statistically significant decrease (*p*-value < 0.01, Dunn’s Test of Multiple Comparisons) in the Hurst exponent in the interval of fractal desynchronization compared to the pre-seizure period. Thus, fractal desynchronization is characterized by an increase in brain effort, which puts the brain in a state of more limited dynamic range [45]. Apparently, this reflects a reduced exchange of information related to fractal dynamics between the EEG sensors in different parts of the brain during desynchronization.

Spectrum power analysis demonstrated that fractal desynchronization is accompanied by an increase in high-frequency and middle-frequency power, which corresponds to the fundamental relation between power and synchrony and manifests itself throughout the human brain [68]. It should be noted the important role played by an increase in the high-frequency power in epileptogenesis and in the initiation of seizures [13,14,68,69]. Apparently, increasing the high-frequency power leads to the deterioration of the relationship between the generalized variations in the analyzed EEG signals on different time scales. These factors significantly degrade the dynamics of self-organizing processes at the onset of an epileptic seizure [11].

The termination of epileptic seizure is accompanied by an increase in fractal synchronization. This result indicates an increased exchange of information related to fractal dynamics in the EEG sensor of the epilepsy focus and other EEG sensors.

As noted in [3], synchronization in epilepsy is a complex phenomenon, and to fully understand this process, there is a need to develop new methods of non-linear analysis.

The proposed method of fractal synchronization is one of the steps in this direction, which makes it possible to study changes in the fractal properties of the brain during an epileptic seizure. We believe that the study of fractal synchronization will provide additional information about the organization of epileptic networks, which would make it possible to contribute to the development of new methods of treatment of epileptic seizures. This approach allows us to reveal a decrease in fractal synchronization at the beginning and an increase in fractal synchronization at the end of an epileptic seizure. This allows this method to be used to detect an epileptic seizure. Perhaps studying the conditions for decreasing or increasing fractal synchronization will make it possible to understand how the mechanisms for stopping an epileptic seizure work.

## Figures and Tables

**Table 1:** Median of the fractal synchronization *FS* for pre-seizure period $FS_{P}$, fractal desynchronization $FS_{D}$, and termination period $FS_{T}$ (*N*—the total number of fractal synchronization values, estimated at the corresponding period; *Me*—median, $Q_{1}$—first quartile, $Q_{3}$—third quartile).

Period	*N*	*FS*, *Me* ($Q_{1}$–$Q_{3}$)
$\mathrm{pre-seizure}\;FS_{P}$	510	0.61 (0.53–0.73)
$\mathrm{desynchronization}\;FS_{D}$	75	0.35 (0.26–0.47)
$\mathrm{termination}\;FS_{T}$	135	0.73 (0.64–0.80)

**Table 2:** The median of the Hurst exponent (*H*) for the pre-seizure period $H_{P}$, for the fractal desynchronization period $H_{D}$, and for the termination period $H_{T}$ (*N*—the total number of the Hurst exponent values, estimated at the corresponding period; *Me*—median; $Q_{1}$—first quartile; $Q_{3}$—third quartile).

Period	*N*	*H*, *Me* ($Q_{1}$–$Q_{3}$)
$\mathrm{pre-seizure}\;H_{P}$	544	0.21 (0.18–0.25)
$\mathrm{desynchronization}\;H_{D}$	80	0.07 (0.04–0.08)
$\mathrm{termination}\;\mathrm{period}\;H_{T}$	144	0.27 (0.21–0.35)

## Data Availability

Dataset available on request from the authors.
